# Quantitative diffusion-weighted MRI response assessment in rhabdomyosarcoma: an international retrospective study on behalf of the European *paediatric* Soft tissue sarcoma Study Group Imaging Committee

**DOI:** 10.1007/s00247-023-05745-z

**Published:** 2023-09-08

**Authors:** Roelof van Ewijk, Cyrano Chatziantoniou, Madeleine Adams, Patrizia Bertolini, Gianni Bisogno, Amine Bouhamama, Pablo Caro-Dominguez, Valerie Charon, Ana Coma, Rana Dandis, Christine Devalck, Giulia De Donno, Andrea Ferrari, Marta Fiocco, Soledad Gallego, Chiara Giraudo, Heidi Glosli, Simone A. J. ter Horst, Meriel Jenney, Willemijn M. Klein, Alexander Leemans, Julie Leseur, Henry C. Mandeville, Kieran McHugh, Johannes H. M. Merks, Veronique Minard-Colin, Salma Moalla, Carlo Morosi, Daniel Orbach, Lil-Sofie Ording Muller, Erika Pace, Pier Luigi Di Paolo, Katia Perruccio, Lucia Quaglietta, Marleen Renard, Rick R. van Rijn, Antonio Ruggiero, Sara I. Sirvent, Alberto De Luca, Reineke A. Schoot

**Affiliations:** 1 Princess Máxima Center for Pediatric Oncology, Heidelberglaan 25, 3584 CS Utrecht, The Netherlands; 2 Image Sciences Institute, UMC Utrecht, Utrecht, The Netherlands; 3 Department of Paediatric Oncology, Children’s Hospital for Wales, University Hospital, Cardiff, UK; 4 Pediatric Hematology-Oncology Unit University-Hospital of Parma, Parma, Italy; 5 Department of Women’s and Children’s Health, University of Padua, Padua, Italy; 6 Pediatric Hematology Oncology Division, University Hospital of Padua, Padua, Italy; 7 Service de Radiologie Interventionnelle Oncologique, Centre Léon Bérard, Lyon, France; 8 Pediatric Radiology Unit, Department of Radiology, Hospital Universitario Virgen del Rocío, Avenida Manuel Siurot S/N, Seville, Spain; 9 Radiology Department, CHU Rennes, Rennes, France; 10 Paediatric Radiology Unit, Vall d´Hebron Hospital Campus, Barcelona, Spain; 11 Department of Hemato-Oncology, HUB, ULB, HUDERF, Brussels, Belgium; 12 Pediatric Oncology Unit, Fondazione IRCCS Istituto Nazionale Tumori, Milan, Italy; 13 Mathematical Institute, Leiden University, Leiden, The Netherlands; 14 Pediatric Oncology Department, Vall d’Hebron Hospital, Barcelona, Spain; 15 Unit of Advanced Clinical and Translational Imaging, Department of Medicine-DIMED, University of Padova, 35122 Padua, Italy; 16 Department of Paediatric Research, Division of Paediatric and Adolescent Medicine, Oslo University Hospital, Oslo, Norway; 17 Department of Radiology and Nuclear Medicine, Wilhelmina Children’s Hospital, UMC Utrecht, Utrecht, The Netherlands; 18 Paediatric Oncology, Cardiff and Vale UHB, Cardiff, UK; 19 Department of Medical Imaging, Radboud University Medical Center, Nijmegen, the Netherlands; 20 Service de Radiothérapie, Centre Eugène Marquis, Rennes, France; 21 Department of Radiotherapy, The Royal Marsden Hospital and The Institute of Cancer Research, Sutton, UK; 22 Department of Radiology, Great Ormond Street Hospital for Children, NHS Foundation Trust, London, UK; 23 Division of Imaging and Oncology, University Medical Center Utrecht, Utrecht University, Utrecht, The Netherlands; 24 Department of Pediatric and Adolescent Oncology, Gustave Roussy Cancer Campus, Université Paris-Saclay, Villejuif, France; 25 Department of Imaging, Institut Gustave Roussy, Villejuif, France; 26 Diagnostic and Interventional Radiology, Fondazione IRCCS Istituto Nazionale Dei Tumori, Milan, Italy; 27 SIREDO Oncology Center (Care, Innovation and Research for Children and AYA With Cancer), Institut Curie, PSL Research University, Paris, France; 28 Department of Radiology and Intervention Unit for Paediatric Radiology, Oslo University Hospital, Ullevål, Norway; 29 Department of Radiology, The Royal Marsden NHS Foundation Trust, London, UK; 30 Department of Radiology, Bambino Gesù Children’s Hospital, IRCCS, Rome, Italy; 31 Pediatric Hematology Oncology, Azienda Ospedaliera Universitaria, Ospedale Santa Maria Della Misericordia, Perugia, Italy; 32 Neuro-Oncology Unit, Department of Paediatric Oncology, Santobono-Pausilipon Children’s Hospital, Naples, Italy; 33 Department of Paediatric Hemato-Oncology, University Hospital Leuven, Louvain, Belgium; 34 Department of Radiology and Nuclear Medicine, Amsterdam UMC Location University of Amsterdam, Amsterdam, the Netherlands; 35 Pediatric Oncology Unit, Fondazione Policlinico Universitario A. Gemelli IRCCS, Università Cattolica del Sacro Cuore, Rome, Italy; 36 Pediatric Radiology Department, Hospital Niño Jesús, Madrid, Spain; 37 Department of Neurology, UMC Utrecht Brain Center, UMC Utrecht, Utrecht, The Netherlands

**Keywords:** Biomarker, Diffusion magnetic resonance imaging, Rhabdomyosarcoma, Sarcoma, Surrogate marker

## Abstract

**Objective:**

To investigate the feasibility of diffusion-weighted magnetic resonance imaging (DW-MRI) as a predictive imaging marker after neoadjuvant chemotherapy in patients with rhabdomyosarcoma.

**Material and methods:**

We performed a multicenter retrospective study including pediatric, adolescent and young adult patients with rhabdomyosarcoma, Intergroup Rhabdomyosarcoma Study group III/IV, treated according to the European *paediatric* Soft tissue sarcoma Study Group (E*p*SSG) RMS2005 or MTS2008 studies. DW-MRI was performed according to institutional protocols. We performed two-dimensional single-slice tumor delineation. Areas of necrosis or hemorrhage were delineated to be excluded in the primary analysis. Mean, median and 5th and 95th apparent diffusion coefficient (ADC) were extracted.

**Results:**

Of 134 included patients, 82 had measurable tumor at diagnosis and response and DW-MRI scans of adequate quality and were included in the analysis. Technical heterogeneity in scan acquisition protocols and scanners was observed. Mean ADC at diagnosis was 1.1 (95% confidence interval [CI]: 1.1–1.2) (all ADC expressed in * 10^−3^ mm^2^/s), versus 1.6 (1.5–1.6) at response assessment. The 5th percentile ADC was 0.8 (0.7–0.9) at diagnosis and 1.1 (1.0–1.2) at response. Absolute change in mean ADC after neoadjuvant chemotherapy was 0.4 (0.3–0.5). Exploratory analyses for association between ADC and clinical parameters showed a significant difference in mean ADC at diagnosis for alveolar versus embryonal histology. Landmark analysis at nine weeks after the date of diagnosis showed no significant association (hazard ratio 1.3 [0.6–3.2]) between the mean ADC change and event-free survival.

**Conclusion:**

A significant change in the 5th percentile and the mean ADC after chemotherapy was observed. Strong heterogeneity was identified in DW-MRI acquisition protocols between centers and in individual patients.

**Graphical Abstract:**

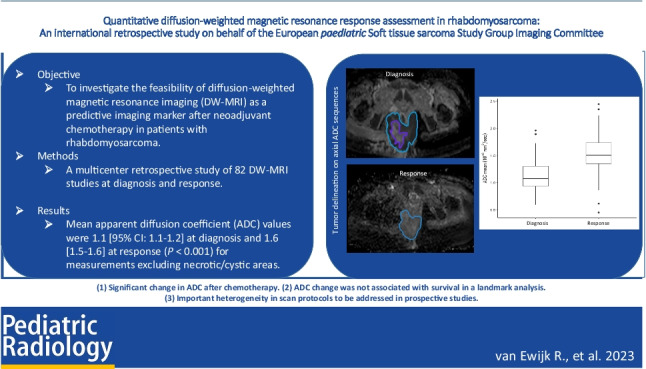

**Supplementary Information:**

Supplementary material is available at 10.1007/s00247-023-05745-z.

## Introduction

Rhabdomyosarcoma is an aggressive soft tissue sarcoma. Currently, there is no reliable biomarker for use as a surrogate endpoint for long-term survival, with clear progression of the primary tumor and development of new lesions being the only features associated with a poorer outcome [1]. Earlier identification of poor or good responders to therapy may support the selection of patients eligible for treatment (de)intensification. Furthermore, it might support earlier evaluation of efficacy of strategies in international phase III studies, which now often require seven to ten years of patient recruitment and data accrual [2, 3].

Diffusion-weighted magnetic resonance imaging (DW-MRI), an imaging modality reflecting the average water displacement in tissues, has become a marker of interest for response assessment in oncology [4]. The apparent diffusion coefficient (ADC), derived from DW-MRI, is a quantification of the degree of free water motion. Tumors with high cellularity have a relative decrease in extracellular volume, which typically results in a decrease in ADC. Histological changes in the tumor, induced by chemotherapy for example, have been linked to changes in ADC, and as such, ADC has been investigated as a response marker in oncology [5, 6]. In preclinical rhabdomyosarcoma models, it has been shown that ADC might be reflective of Ki67 proliferation indices [7] and that therapy-induced tumor necrosis or growth corresponds with increases and decreases in ADC values, respectively [8]. However, the ADC data from current clinical studies [9–12] are insufficient for clinical implementation and do not adequately address factors potentially contributing to measurement variability, as reported in other tumor types [4].

In this study, we aimed to investigate the feasibility of DW-MRI in patients with rhabdomyosarcoma as a marker of response to neoadjuvant chemotherapy. As DW-MRI involves a number of technical choices and processing steps, we evaluated the variability in DW-MRI acquisition protocols. An understanding of the variability, both technical and between patients, is essential to inform future prospective studies, because phase III studies in this rare tumor require the participation of over 100 hospitals. As such, the primary objectives of this retrospective study were to assess the degree of ADC change after chemotherapy; to describe the applied DW-MRI acquisition protocols; and to evaluate the impact of tumor segmentation variability on measured ADC values. Secondary objectives were to evaluate the association between ADC values and survival. The results of this feasibility study will be used to improve the methodology to accurately acquire, estimate and analyze DW-MRI markers in rhabdomyosarcoma.

## Methods

### Participant selection

Eligible patients were retrospectively selected from participating sites of the European *paediatric* Soft tissue sarcoma Study Group (E*p*SSG) RMS2005 and MTS2008 studies. The E*p*SSG RMS2005 and MTS2008 studies were approved by institutional review boards and all patients and/or parents gave written informed consent. Patients from The Netherlands, treated according to the E*p*SSG RMS2005 and MTS2008 protocols but not included in the study, signed informed consent as approved by the responsible authority. This retrospective study was approved by the medical research ethics committee (UMC Utrecht, reference-ID: 18–412).

Pediatric, adolescent and young adult patients, between 6 months and 21 years of age, with either localized or metastatic Intergroup Rhabdomyosarcoma Study (IRS) group III/IV histologically-proven rhabdomyosarcoma who were treated according to the E*p*SSG RMS2005 (ClinicalTrials.gov identifier: NCT00379457) or E*p*SSG MTS2008 study (ClinicalTrials.gov identifier: NCT00379457) protocols with available DW-MRI scans were eligible. The E*p*SSG RMS2005 study was an academic, international, randomized, phase III trial, open from 2006 to 2016 including patients with localized rhabdomyosarcoma [2, 3]. The E*p*SSG MTS2008 study was an academic, international, prospective study, open from 2010 to 2016, including patients with metastatic rhabdomyosarcoma [13]. Survival was updated after closure of the studies. Participating centers were selected by the study national coordinators.

### Imaging protocols

The study protocols included basic recommendations for MRI, without any specific guidance for DW-MRI sequences. All institutional MRI protocols were accepted. The baseline MRI was performed within 28 days of initiation of treatment. Early response evaluation per protocol was obtained after three 3-weekly cycles of chemotherapy. In case of protocol non-adherence, scans after two or four cycles with available DW-MRI were accepted.

### Data collection and quality assessment

De-identified MRI data were collected on a platform developed as part of the Quality and Excellence in Radiotherapy and Imaging for Children and Adolescents with Cancer across Europe in Clinical Trials initiative of the European Society for Paediatric Oncology (SIOP Europe) [14]. MRI data were extracted and analyzed using an in-house program. Selected Digital Imaging and Communications in Medicine parameters essential for evaluation of DW-MRI technical variance (e.g., MRI vendor, TE, number of diffusion weightings (*B* values), maximum *B* value, echo time) were extracted. Intra-individual comparison of scan parameters between diagnosis and response was performed for heterogeneity; for continuous markers we considered parameters within a range of 10% between diagnosis and response as homogeneous. The diagnostic quality of each MRI was recorded by two pediatric radiologists (S.H. and R.R., with 10 and 18 years of experience in pediatric musculoskeletal radiology, respectively). A semi-quantitative scale, ranging from 1 to 3, was applied: 1 = poor, not evaluable (i.e. significant artefact); 2 = moderate, evaluable; 3 = good. Scans of poor quality were excluded from the analysis.

### Tumor delineation

**Fig. 1:**
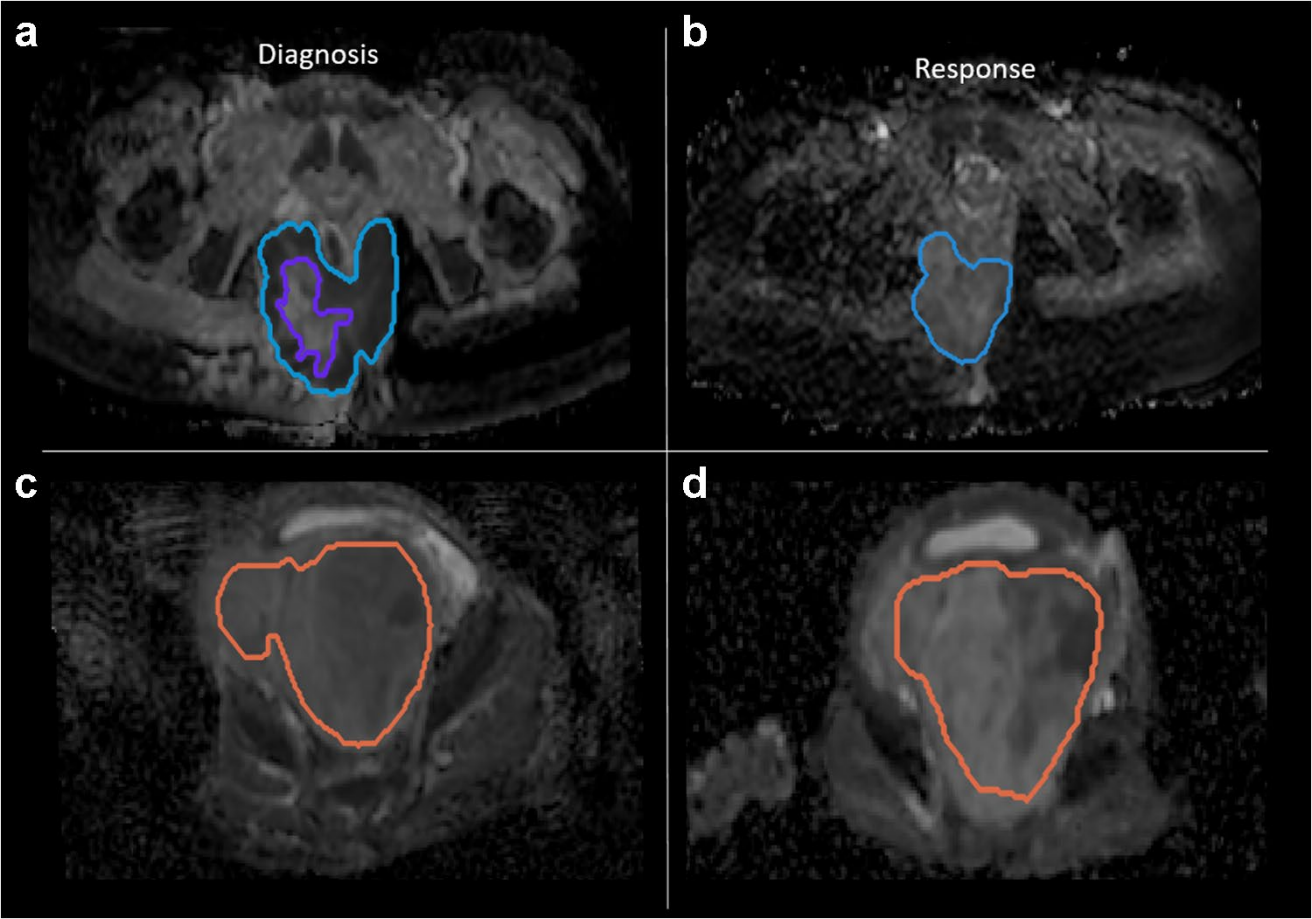
Tumor segmentation of diagnosis (**a**, **c**) and response (**b**, **d**) axial diffusion-weighted MRI (apparent diffusion coefficient) scans. **a**, **b** A 16-year-old girl with a perianal alveolar rhabdomyosarcoma. The whole tumor (*blue outline*) is delineated. The hemorrhagic component (*inner purple outline* in **a**) was excluded from the analysis. **c**, **d** A 1-year-old boy with a retroperitoneal pelvic rhabdomyosarcoma. The whole tumor (*red outline*) is delineated

All scans were evaluated by one pediatric radiologist (S.H. or R.R.). Anatomical imaging (T1, T2, post-contrast T1) was reviewed and two-dimensional tumor segmentation on a single axial DW-MRI slice was performed. A randomly selected subset of 20 patients were segmented by both radiologists for assessment of inter-observer variability, where the second delineation was performed on the same tumor slice. Investigators were blinded to patient characteristics and outcome. Single-slice segmentation was performed on the axial image with the largest proportion of homogeneous tumor. The inner edge of the tumor was delineated to minimize the risk of including peritumoral edema or adjacent tissues in the region-of-interest (ROI). Secondly, intralesional necrotic and cystic areas and artifacts were delineated (Fig. 1). For primary analysis, hemorrhage, cystic parts and artefacts were excluded.

### Parameters

ADC was calculated from DW-MRI data where available. In the absence of raw DW-MRI data, ADC maps were used. The following ADC measures were extracted: mean, median, 5th percentile and 95th percentile. The choice for the 5th and 95th percentiles was made to reduce aberrant measures of minimal or maximum ADC due to artifacts. As such, we considered the 5th percentile an optimal measure of low ADC values.

### Statistical analysis

The primary outcome of this study was the absolute change in mean ADC at the early response evaluation. ADC at diagnosis and early response were reported as secondary outcomes. ADC measures were compared between baseline and response using the paired *t*-test. The relation between stratifying patient and tumor characteristics (age, tumor size, E*p*SSG RMS2005 risk group [2, 3]) with ADC values at baseline or response was examined using the independent Student’s *t*-test. For characteristics with more than two categories, an ANOVA was performed with Tukey’s post hoc analysis. ADC measures were evaluated for the definition of the ROI and compared with paired *t*-test. We measured the inter-observer variability using the intraclass correlation coefficient for single measurements.

We evaluated the relation between ADC measures and event-free survival (EFS). An event was defined as disease progression, recurrence, or death due to any cause. A waterfall plot for the distribution of mean ADC change was used to visualize mean ADC change corresponding to the event status. Univariable Cox proportional hazard regression models were used to estimate the association between the ADC measures and EFS. For the analysis of mean ADC at baseline, the date of diagnosis was used. A landmark analysis at nine weeks after the date of diagnosis was used to estimate the association between change in mean ADC, mean ADC at response, and EFS [15, 16]. All statistical analyses were performed with R software version 4.1.1 [17].

## Results

### Patient characteristics

We enrolled 134 patients from seven countries (Belgium 10 patients; France 16; Italy 36; Norway 12; Spain 10; The Netherlands 46; UK 4). Median age was 6.0 years (range 0.3–21.8). Almost three-quarters of the patients had an embryonal rhabdomyosarcoma, nearly a quarter an alveolar rhabdomyosarcoma. Localized and metastatic disease were seen in 80% and 20% of patients, respectively (Table 1).

**Table 1:** Patient and tumor characteristics

	Included patients (*n*=82)	Excluded patients (*n*=52)
Sex		
Female	22 (26.8%)	26 (50.0%)
Male	60 (73.2%)	26 (50.0%)
Age (years)		
Median [Min, Max]	5.9 [0.3, 21.8]	6.3 [0.6, 21.7]
Site of primary tumor		
Extremities	6 (7.3%)	6 (11.5%)
GUBP	18 (22.0%)	6 (11.5%)
GUnoBP	2 (2.4%)	1 (1.9%)
HNnoPM	10 (12.2%)	7 (13.5%)
HNPM	29 (35.4%)	18 (34.6%)
Orbit	9 (11.0%)	8 (15.4%)
Other site	8 (9.8%)	6 (11.5%)
Histology		
Alveolar	14 (17.1%)	15 (28.8%)
Embryonal	64 (78.0%)	32 (61.5%)
Other	4 (4.9%)	5 (9.6%)
Fusion status		
Negative	51 (62.2%)	27 (51.9%)
Positive	11 (13.4%)	10 (19.2%)
Missing	20 (24.4%)	15 (28.8%)
Tumor size		
≤ 5 cm	39 (47.6%)	31 (59.6%)
> 5 cm	43 (52.4%)	21 (40.4%)
Tumor stage		
T0	0 (0%)	1 (1.9%)
T1	31 (37.8%)	31 (59.6%)
T2	51 (62.2%)	20 (38.5%)
Nodal stage		
N0	58 (70.7%)	36 (69.2%)
N1	24 (29.3%)	16 (30.8%)
Risk group		
Standard	27 (32.9%)	20 (38.5%)
High	33 (40.2%)	18 (34.6%)
Very high	7 (8.5%)	2 (3.8%)
Metastatic	15 (18.3%)	12 (23.1%)

*GUBP* genitourinary bladder prostate, *GUnoBP *genitourinary non-bladder prostate, *HNPM* head neck parameningeal, *HNnoPM* head neck non-parameningeal, *Max* maximum, *Min* minimum

### Diffusion-weighted magnetic resonance imaging quality assessment

**Fig. 2:**
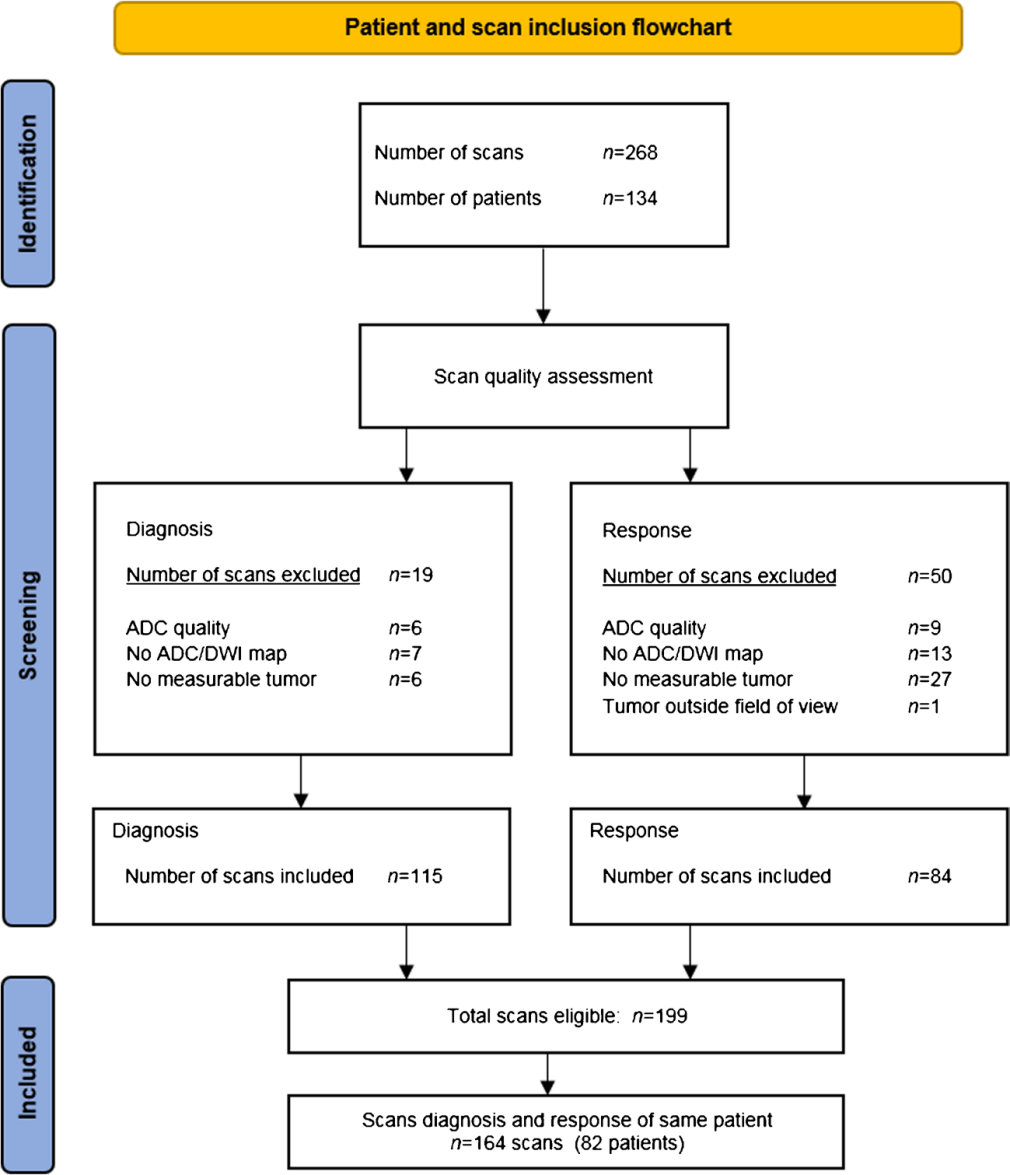
Patient and scan selection. *ADC* apparent diffusion coefficient, *DWI* diffusion-weighted imaging

In total, 268 scans were uploaded. After quality control, 199 scans were considered eligible. Reasons for exclusion were insufficient quality (*n*=15), no available DW-MRI scans (*n*=20), no measurable tumor (*n*=33) and tumor outside the field of view (*n*=1). The DW-MRI scans of 82 patients at diagnosis and at early response were included for analysis (Fig. 2).

### Magnetic resonance imaging acquisition characteristics

Of 268 evaluated scans, 19 had no general scan characteristics available and 24 were without specific diffusion characteristics (Table 2). Intra-individual comparison of scans showed that 14 patients had a different MRI manufacturer at diagnosis than at response (Supplementary Material 1). For the other selected parameters, comparison between diagnosis and response showed the average slice thickness to be more than 10% different in 26 patients; pixel spacing differed in 29 patients by more than 10%; and the mean echo time differed in 24 patients by more than 10%. In only 38 patients (46%) were technical parameters at diagnosis and at treatment response similar (Supplementary Material 2).

**Table 2:** Diffusion-weighted magnetic resonance imaging acquisition characteristics

	Full cohort (*n*=268)
Manufacturer	
GE, Healthcare Technologies, Waukesha, WI, USA	23 (8.6%)
Philips, Best, The Netherlands	117 (43.7%)
Siemens, Erlangen, Germany	109 (40.7%)
Not available	19 (7.1%)
Slice thickness (mm)	
Mean (SD)	4.30 (0.975)
Median [Min, Max]	4.00 [2.00, 7.00]
Not available	19 (7.1%)
Number of *B* values	
Mean (SD)	2.93 (1.52)
Median [Min, Max]	2.00 [2.00, 10.0]
Not available	24 (9.0%)
Pixel spacing (mm)	
Mean (SD)	1.33 (0.448)
Median [Min, Max]	1.25 [0.332, 2.73]
Not available	19 (7.1%)
Highest *B* value	
Mean (SD)	962 (89.3)
Median [Min, Max]	1000 [800, 1400]
Not available	24 (9.0%)
Echo time (ms)	
Mean (SD)	78.0 (13.6)
Median [Min, Max]	77.8 [30.0, 134]
Not available	19 (7.1%)

*Max *maximum,* Min *minimum,* SD* standard deviation

### Apparent diffusion coefficient measurements

**Fig. 3:**
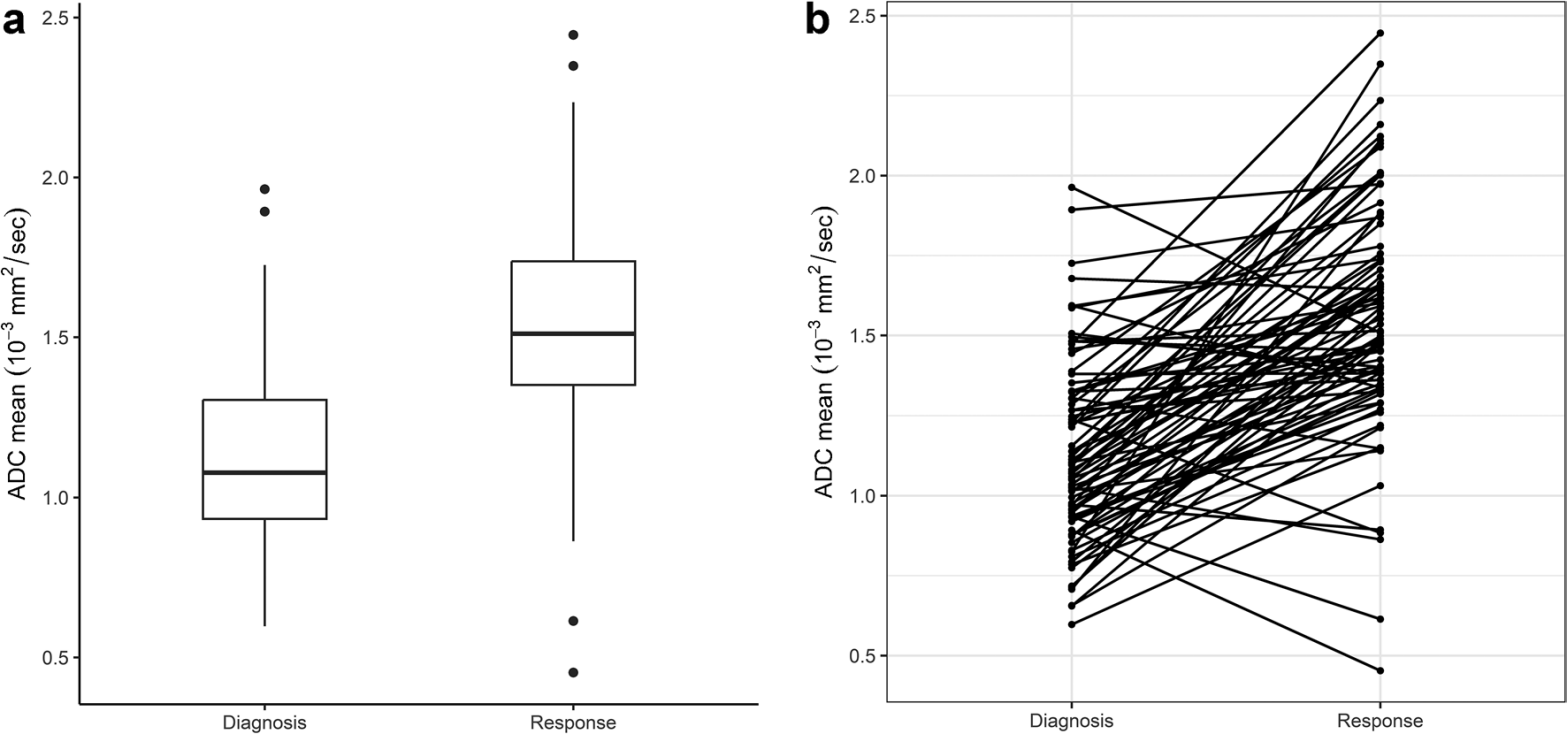
Mean apparent diffusion coefficient (ADC) parameters excluding necrotic/cystic areas. **a** Boxplot shows values at diagnosis and response. **b** Graph shows individual changes in mean ADC at diagnosis and response

The mean ADC values were 1.1 (95% confidence interval [CI]: 1.1–1.2) at diagnosis and 1.6 (1.5–1.6) at response (*P*< 0.001), for measurements excluding necrotic/cystic areas (Fig. 3). The mean absolute ADC change after neoadjuvant chemotherapy was 0.4 (0.3–0.5) and the mean percentage change was 44% (35–54). The mean of the median ADC was 1.1 (1.0–1.2) at diagnosis and 1.6 (1.5–1.7) at response (*P*< 0.001). The median absolute ADC change was 0.5 (0.4–0.6) with an average median percentage change of 50% (39–61). The 5th percentile ADC was 0.8 (0.7–0.9) at diagnosis and 1.1 (1.0–1.2) at response (*P*< 0.001). The 95th percentile ADC was 1.6 (1.5–1.6) at diagnosis and 2.0 (1.9–2.1) at response (*P*< 0.001) (Table 3).

**Table 3:** Apparent diffusion coefficient values (95% confidence interval) based on tumor characteristics

ADC	Diagnosis	Response	Absolute change	Percentage change
Excluding necrotic/cystic areas				
Mean	1.13 (1.07–1.19)	1.55 (1.47–1.63)	0.42 (0.34–0.51)	44.2 (34.6–53.9)
Median	1.10 (1.04–1.17)	1.56 (1.48–1.65)	0.46 (0.37–0.56)	50.1 (39.1–61.2)
5th percentile	0.80 (0.74–0.86)	1.07 (0.98–1.15)	0.26 (0.18–0.34)	
95th percentile	1.55 (1.48–1.63)	2.01 (1.92–2.10)	0.46 (0.36–0.56)	
Including necrotic/cystic areas				
Mean	1.13 (1.07–1.19)	1.56 (1.48–1.64)	0.43 (0.34–0.51)	44.3 (34.6–54.0)
Median	1.10 (1.04–1.17)	1.57 (1.48–1.66)	0.47 (0.37–0.56)	50.4 (39.3–61.4)
5th percentile	0.80 (0.74–0.86)	1.06 (0.98–1.15)	0.26 (0.18–0.35)	
95th percentile	1.56 (1.48–1.64)	2.02 (1.93–2.11)	0.46 (0.36–0.56)	
Alveolar rhabdomyosarcoma				
Mean	0.96 (0.78–1.13)	1.42 (1.26–1.59)	0.46 (0.24–0.69)	55.4 (33.4–77.4)
Median	0.90 (0.73–1.07)	1.44 (1.25–1.62)	0.53 (0.30–0.77)	68.2 (40.5–95.9)
Embryonal rhabdomyosarcoma				
Mean	1.17 (1.11–1.24)	1.57 (1.48–1.67)	0.40 (0.30–0.50)	39.7 (28.6–50.9)
Median	1.16 (1.09–1.23)	1.58 (1.48–1.68)	0.42 (0.31–0.53)	43.5 (31.1–55.8)
Tumor size ≤ 5 cm				
Mean	1.11 (1.02–1.20)	1.54 (1.41–1.68)		
Median	1.10 (1.01–1.19)	1.56 (1.42–1.70)		
Tumor size > 5 cm				
Mean	1.15 (1.05–1.24)	1.56 (1.47–1.66)		
Median	1.11 (1.01–1.20)	1.57 (1.46–1.67)		
Standard risk group				
Mean	1.17 (1.06–1.28)	1.51 (1.33–1.69)		
Median	1.17 (1.05–1.28)	1.53 (1.34–1.72)		
High-risk group				
Mean	1.19 (1.07–1.30)	1.60 (1.51–1.70)		
Median	1.16 (1.04–1.28)	1.62 (1.51–1.72)		
Very high–localized				
Mean	0.85 (0.78–0.91)	1.65 (1.26–2.04)		
Median	0.77 (0.70–0.84)	1.69 (1.24–2.13)		
Very high–metastatic				
Mean	1.05 (0.94–1.16)	1.47 (1.28–1.66)		
Median	1.01 (0.91–1.12)	1.46 (1.26–1.66)		

*ADC* apparent diffusion coefficient

### Apparent diffusion coefficient measurements, including necrotic/cystic regions

Mean ADC, including necrotic/cystic regions, was 1.1 (1.1–1.2) at diagnosis and 1.6 (1.5–1.6) at early response, which was not significantly different when compared to the mean ADC of ROI excluding these areas (*P*=0.1 and *P*=0.42, respectively). Absolute mean ADC change was 0.4 (0.3–0.5) and percentage ADC change was 44% (35–54), which was not significantly different to measurements excluding necrotic/cystic areas (*P*=0.80 and *P*=0.81, respectively), which was also observed in sub-analysis of patients with homogeneous scanning properties at diagnosis and response (Supplementary Material 3).

**Fig. 4:**
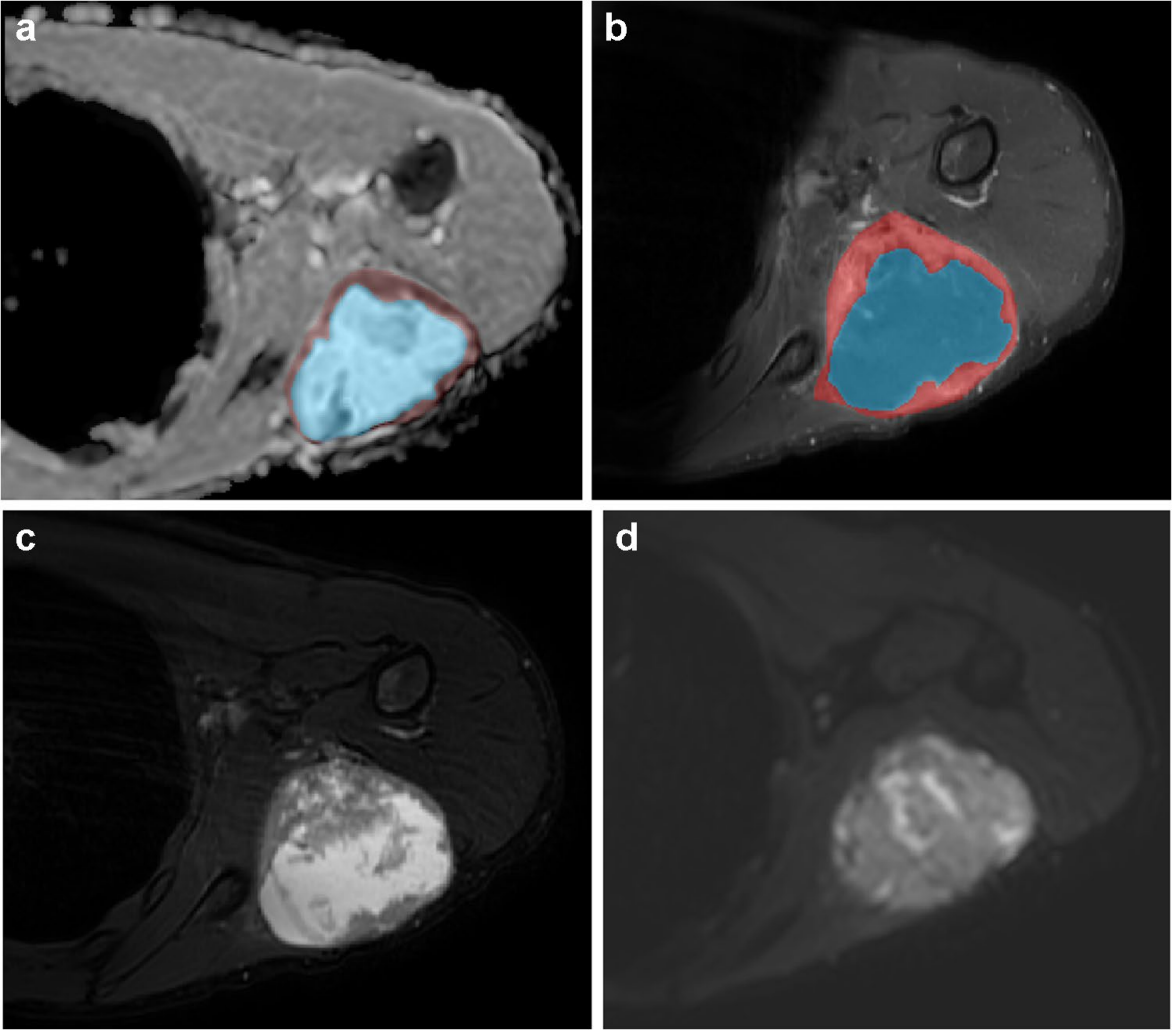
A 14-year-old boy with an embryonal rhabdomyosarcoma of the left upper extremity, located in the teres minor, with central necrosis. Axial apparent diffusion coefficient (ADC) (**a**), T1 post-contrast (**b**), T2 (**c**) and diffusion-weighted (**d**) images. The mean ADC was 71% higher when including compared to excluding the necrotic region. *Blue* intra-tumoral hemorrhage, *brown/red* tumor tissue

In subgroup analyses of all patients with necrotic/cystic areas delineated (nine at diagnosis and five at response), the mean ADC for scans including necrosis was on average 8% higher (range; 8% to 71%). Most scans, 12 out of 14, had a mean ADC difference variability within 10% when comparing ROIs with or without necrotic/cystic areas. There was one outlier, a diagnostic study of an embryonal rhabdomyosarcoma of the extremity with a large area of necrosis and a mean ADC of 2.3 versus 1.4 (excluding the necrotic region) (Fig. 4).

### Apparent diffusion coefficient measurements for patient and tumor characteristics

In subgroup analysis of pediatric and adolescent patients up to 18 years of age (baseline characteristics in Supplementary Material 4 and 5), the mean ADC values of pediatric and adolescent patients were 1.1 (95% CI: 1.1–1.2) at diagnosis and 1.6 (1.5–1.6) at response. The mean absolute ADC change after neoadjuvant chemotherapy was 0.4 (0.3–0.5) and the mean percentage change was 45% (35–55). ADC values of the pediatric and adolescent patients were not significantly different as compared to the whole cohort (Supplementary Material 6). Direct comparison of ADC values of pediatric and adolescent patients (*n*=81) versus young adult patients (*n*=1) was not feasible.

**Fig. 5:**
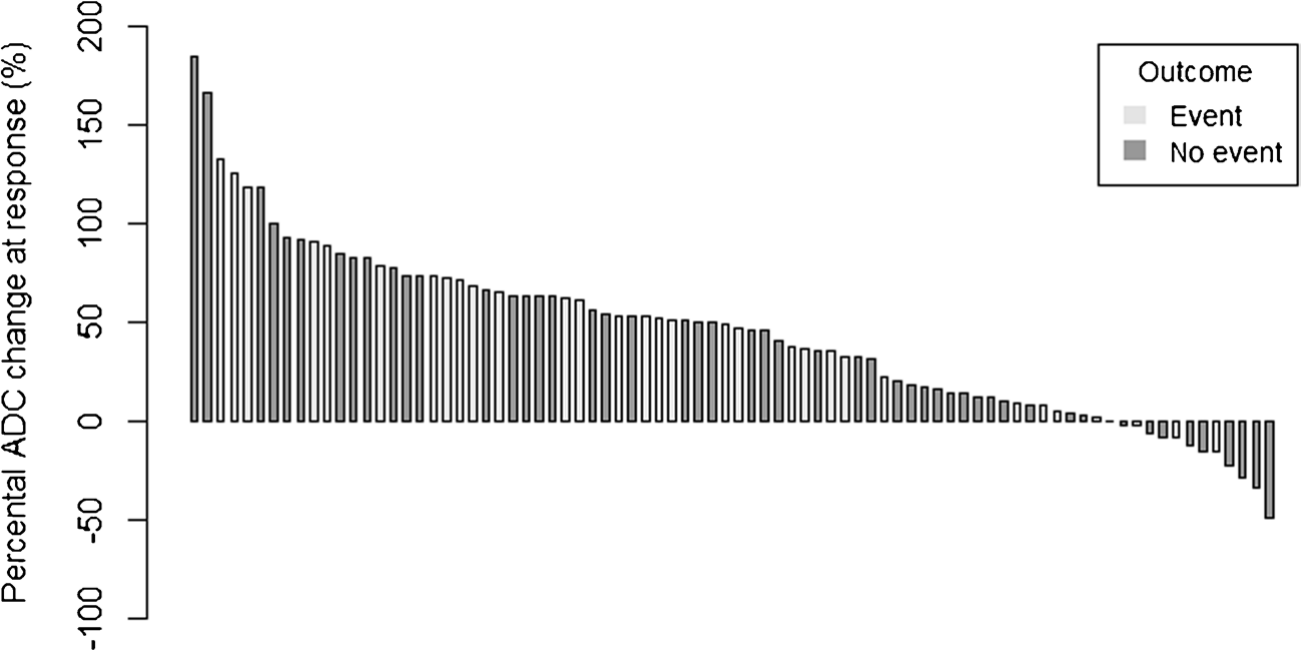
Waterfall plot showing mean apparent diffusion coefficient (ADC) percentage change per patient for patients with and without a tumor-related event

For alveolar rhabdomyosarcoma (*n*=14), mean and median ADC at diagnosis were 1.0 (0. 8–1.1) and 0.9 (0.7–1.1) versus 1.4 (1.3–1.6) and 1.4 (1.3–1.6) at response. For embryonal rhabdomyosarcoma (*n*=64), mean and median ADC at diagnosis, 1.2 (1.1–1.2) and 1.2 (1.1–1.2), respectively, were significantly higher compared to ADC in tumors with alveolar histology (*P*=0.02 and *P*=0.01). At response, mean and median of embryonal histology, 1.6 (1.5–1. 7) and 1.6 (1.5–1.7), respectively, were not significantly different from alveolar histology (*P*=0.11 and *P*=0.16). Absolute change in mean ADC was 0.5 (0.2–0.7) for alveolar histology and 0.4 (0.3–0.5) for embryonal histology (*P*=0.55).

For tumors larger than 5 cm at diagnosis, mean and median ADC were 1.2 (1.1–1.2) and 1.1 (1.0–1.2), respectively at diagnosis versus 1.6 (1.5–1.7) and 1.6 (1.5–1.7), respectively at response. For tumors of 5 cm or smaller at diagnosis, mean and median ADC were 1.1 (1.0–1.2) and 1.1 (1.0–1.2), respectively at diagnosis versus 1.5 (1.4–1.7) and 1.6 (1.4–1.7), respectivley at response. ADC measurements were not significantly different for tumor size at diagnosis.

ANOVA of mean and median ADC for treatment risk group showed a significant difference at diagnosis. No significant differences for risk group at response were identified. Tukey’s post hoc test showed a significant difference in mean and median ADC at diagnosis between the very high–localized risk group versus the standard risk group (*P*=0.03 and *P*=0.01) and the high-risk group (*P*=0.02 and *P*=0.01). ADC mean and median in the very high–localized group at diagnosis were 0.9 (0.8–0.9) and 0.8 (0.7–0.8), respectively. ADC mean and median were 1.2 (1.1–1.3) and 1.2 (1.1–1.3) in the standard risk group, 1.2 (1.1–1.3) and 1.2 (1.0–1.3) in the high-risk group, and 1.1 (0.9–1.2) and 1.0 (0.9–1.1) in the very high–metastatic group, respectively (Table 3).

### Apparent diffusion coefficient measurements for survival

The estimated hazard ratio from the univariable Cox hazard regression model showed no association at baseline between ADC 5th percentile (HR 95% CI: 0.2–2.6) or mean ADC (HR 95% CI: 0.1–1.6) and EFS. No association of ADC 5th percentile (HR 95% CI: 0.5–3.1) or mean ADC (HR 95% CI: 0.4–2.3) at response and absolute change in ADC 5th percentile (HR 95% CI: 0.61–3.9) or mean ADC (HR 95% CI: 0.6–3.2) and EFS was observed at the landmark point (Table 4, Fig. 5). Sub-analysis of the cohort with homogeneous scanning properties at diagnosis and response showed similar results (Supplementary Material 7).

**Table 4:** Univariable Cox proportional hazard analysis for event-free survival

Variable		Hazard ratio	(95% CI)
ADC 5th percentile	Absolute change	1.5	(0.61–3.9)
Mean ADC	Absolute change	1.3	(0.57–3.2)
ADC 5th percentile	Diagnosis	0.72	(0.2–2.6)
Mean ADC	Diagnosis	0.43	(0.11–1.6)
ADC 5th percentile	Response	1.3	(0.52–3.1)
Mean ADC	Response	0.92	(0.37–2.3)

*ADC* apparent diffusion coefficient, *CI* confidence interval

### Inter-observer variability

For inter-observer analysis, 20 patients were randomly selected. Intraclass correlation for mean ADC between two readers for selected slice delineation was 0.93 (95% CI: 0.83–0.97) for diagnosis and 0.96 (0.90–0.99) for response.

## Discussion

This study shows a significant change in ADC 5th percentile, mean and median values of the primary tumor at response assessment after three cycles of chemotherapy. DW-MRI acquisition protocols showed high heterogeneity in and among individuals when comparing scans at diagnosis and response. Exploratory analyses of mean ADC revealed a significant difference for tumor histology and risk group status at baseline. Univariable Cox regression analysis did not show an association between the change in the ADC 5th percentile or mean ADC and EFS. Analysis of inter-observer variability in a selected group exhibited excellent agreement.

The change in ADC after chemotherapy identified in this study is in line with preclinical research [7]. However, whereas in other solid cancers, like brain [18] and breast tumors [19, 20], DW-MRI has become standard in diagnostic and response imaging, studies in rhabdomyosarcoma are thus far mainly focused on diffusion measurements at presentation to narrow the differential diagnosis of a soft tissue mass [5, 21]. Available reports have mainly focused on patients with head-neck rhabdomyosarcoma [9]. As such, comparative studies for this work in rhabdomyosarcoma, as for soft tissue sarcoma, are limited. The prognostic value of baseline ADC and diffusion restrictive volume in children and adolescents with head-neck rhabdomyosarcoma has been described in one retrospective cohort [11]. Although the included cohort differed in tumor location and age compared to this study, the mean reported ADC of 1.04 [11] is in a similar range to our observation of mean ADC at diagnosis. The authors concluded that lower ADC at baseline might correlate with overall survival, which in our view might be explained by alveolar histology of six patients in the study, given that in our study we observed lower mean and median ADC values for patients with alveolar compared to embryonal rhabdomyosarcoma. However, it is unclear what the underlying biological explanation is. The question of whether low mean ADC at diagnosis is an independent risk factor needs to be investigated including in the analysis known risk factors such as histology and fusion status, for localized and metastatic rhabdomyosarcoma [13, 22–24].

In our study, we describe the heterogeneity of DW-MRI acquisition parameters, as it is reported to be an important source of variability for quantitative applications. In the literature, the underlying tumor biology, the scan operator, the hardware and software of the MRI system, including the DW-MRI acquisition protocol, the algorithm to convert DW-MRI to ADC and definition of ROIs are considered to be the most important factors leading to variability [4]. In our study, DW-MRI systems and acquisition protocols were frequently different within individuals, explained in several ways. First, frequently an MRI is performed before referral to a tertiary center and is not always repeated. Second, due to the rarity of the disease, scan operators might not be familiar with soft tissue sarcoma-specific protocols. This is complicated by the fact that rhabdomyosarcoma may occur anywhere in the body, and thus different scanning protocols, specific for body sites, are in practice. Lastly, we observed that the raw DW-MRI data were not always stored, which limited our ability to recalculate the ADC independent of the system software. As only 46% of the included cohort of our study had similar DW-MRI parameters, technical variability is an important subject in this and for future studies in this tumor.

We evaluated the difference between two different ROIs. In the literature, a wide methodological variety in the definition of ROIs is described in sarcoma [25]. A proof-of-concept study showed higher ADC measurements when including necrotic or cystic areas [25]. Although we did not observe a significant difference in ADC values, on an individual level, potentially relevant differences were identified when validating ADC as an individual response marker to therapy. Investigating the measurement variability caused by technical factors is an interesting topic for further research.

In our study, we present a cohort of rhabdomyosarcoma patients who underwent DW-MRI. Multiple limitations are important to acknowledge. In 20% of the eligible patients, early response assessment after three cycles was not possible due to the lack of measurable tumor. This complicates the clinical validation and implementation of DW-MRI as a response marker, as patients with complete remission (non-measurable disease) at early response evaluation were not reported to be a prognostic subgroup [1, 26]. Furthermore, due to lack of MRI standardization, high heterogeneity was observed in this retrospective study, which limits the validity of our results.

To improve quantitative DW-MRI studies, we will need to evaluate the magnitude of the impact of technical variability on ADC measurements. It will be essential to investigate methods for optimal procedures in data acquisition and quality control and assurance for harmonization and standardization of DW-MRI data to be representative and of diagnostic quality, as, for example, performed in quantitative fluorodeoxyglucose-positron emission tomography imaging by the European Association of Nuclear Medicine [1, 27, 28]. To raise awareness and improve protocol adherence, a European rhabdomyosarcoma imaging guideline was developed in a multi-organizational collaboration, including technical MRI protocols [29]. For validation and trial design of quantified imaging biomarkers, the Quantitative Imaging Biomarkers Alliance of the Radiological Society of North America and the European Imaging Biomarker Alliance of the European Society of Radiology provide guidance for methodological standards [30–32], which will be incorporated in the upcoming prospective study.

In conclusion, we have demonstrated the feasibility of ADC measurement in rhabdomyosarcoma and highlight important methodological considerations to take forward in prospective assessments of the predictive value of DW-MRI as a response marker.

## Supplementary Information

Below is the link to the electronic supplementary material.

Supplementary file1 (DOCX 51.0 KB)

## Data Availability

The datasets generated during and/or analyzed during the current study are available from the corresponding author on reasonable request.
