# Supplementary material for: Quantitative diffusion-weighted MRI response assessment in rhabdomyosarcoma: an international retrospective study on behalf of the European paediatric Soft tissue sarcoma Study Group Imaging Committee
Source: Pediatr Radiol. 2023 Sep 8;53(12):2539–51. doi: 10.1007/s00247-023-05745-z (PMC10635937; doi:10.1007/s00247-023-05745-z)
Supplement: Supplementary file 1 — Supplementary file1 (DOCX 51.0 KB) [file 247_2023_5745_MOESM1_ESM.docx]

Quantified diffusion-weighted MR imaging response assessment in rhabdomyosarcoma: an international retrospective study on behalf of the EpSSG Imaging Committee

Supplemental file

Supplementary Material 1. DW-MRI acquisition characteristics for the excluded and included cohort

Supplementary Material 2. Evaluation of homogeneous, intra-individual, scan parameters between diagnosis and response

Supplementary Material 3. Comparison for including versus excluding necrotic areas for patients with homogeneous scanner properties at diagnosis and response (*n*=38)

Supplementary Material 4.

Supplementary Material 5. Baseline characteristics of the pediatric/adolescent versus young adult cohort in the excluded and included cohort

Supplementary Material 7. Univariable Cox proportional hazard analysis for event-free survival for patients with homogeneous scanner properties at diagnosis and response (*n*=38)

Supplementary Material 8. Tumor related events in the full and paired cohort

| Suppl. Material 1. DW-MRI acquisition characteristics for the excluded and included cohort | | |  |
| --- | --- | --- | --- |
|  | Excluded (N=104) | Included |  |
| Manufacturer |  |  |  |
| GE, Healthcare Technologies, Waukesha, WI, USA | 7 (6.7%) | 16 (9.8%) |  |
| Philips, Best, The Netherlands | 44 (42.3%) | 73 (44.5%) |  |
| Siemens, Erlangen, Germany | 34 (32.7%) | 75 (45.7%) |  |
| Not available | 19 (18.3%) | 0 (0%) |  |
| Slice thickness (mm) |  |  |  |
| Mean (SD) | 4.26 (1.05) | 4.32 (0.939) |  |
| Median [Min, Max] | 4.00 [2.00, 7.00] | 4.25 [2.00, 7.00] |  |
| Not available | 19 (18.3%) | 0 (0%) |  |
| Number of B values |  |  |  |
| Mean (SD) | 2.79 (1.27) | 2.99 (1.63) |  |
| Median [Min, Max] | 2.00 [2.00, 10.0] | 2.00 [2.00, 10.0] |  |
| Not available | 22 (21.2%) | 2 (1.2%) |  |
| Pixel spacing (mm) |  |  |  |
| Mean (SD) | 1.26 (0.456) | 1.37 (0.441) |  |
| Median [Min, Max] | 1.19 [0.332, 2.73] | 1.37 [0.449, 2.73] |  |
| Not available | 19 (18.3%) | 0 (0%) |  |
| Highest B value |  |  |  |
| Mean (SD) | 954 (101) | 965 (82.7) |  |
| Median [Min, Max] | 1000 [800, 1400] | 1000 [800, 1200] |  |
| Not available | 22 (21.2%) | 2 (1.2%) |  |
| Echo time (ms) |  |  |  |
| Mean (SD) | 78.1 (15.5) | 77.9 (12.6) |  |
| Median [Min, Max] | 75.6 [30.0, 134] | 78.0 [52.0, 109] |  |
| Not available | 19 (18.3%) | 0 (0%) |  |
|  | | | |

| Suppl. Material 2. Evaluation of homogeneous, intra-individual, scan parameters between diagnosis and response | | |
| --- | --- | --- |
|  | All patients  (*n*=134) | Included for ADC analyses  (*n*=82) |
| Manufacturer |  |  |
| Equal | 96 (71.6%) | 68 (82.9%) |
| Different | 38 (28.4%) | 14 (17.1%) |
| Slice thickness (mm)* |  |  |
| Equal | 71 (53.0%) | 56 (68.3%) |
| Different | 63 (47.0%) | 26 (31.7%) |
| Number of B values |  |  |
| Equal | 88 (65.7%) | 66 (80.5%) |
| Different | 46 (34.3%) | 16 (19.5%) |
| Pixel spacing (mm)* |  |  |
| Equal | 70 (52.2%) | 53 (64.6%) |
| Different | 64 (47.8%) | 29 (35.4%) |
| Highest B value* |  |  |
| Equal | 92 (68.7%) | 71 (86.6%) |
| Different | 42 (31.3%) | 11 (13.4%) |
| Echo time (ms)* |  |  |
| Equal | 81 (60.4%) | 58 (70.7%) |
| Different | 53 (39.6%) | 24 (29.3%) |
| All combined |  |  |
| Full scan equal | 44 (32.8%) | 38 (46.3%) |
| Different | 90 (67.2%) | 44 (53.7%) |
| * A 10% range of difference between scans was considered homogeneous | | |

| Suppl. Material 3. Comparison for including versus excluding necrotic areas for patients with similar scanner properties at diagnosis and response (*n*=38) | | | |
| --- | --- | --- | --- |
| Variable | Excluding | Including | P value |
| Mean ADC diagnosis | 1.1 | 1.1 | 0.13 |
| Mean ADC response | 1.496 | 1.501 | 0.32 |
| Mean ADC percental change | 41.98 % | 42.09 % | 0.75 |
| ADC: Apparent Diffusion Coefficient | | | |

Suppl. Material 4. Baseline characteristics of pediatric/adolescent versus young adult cohort

|  | Pediatric/Adolescent (*n*=129) | Young adult (*n*=5) | *P*-value |
| --- | --- | --- | --- |
| Country |  |  |  |
| France | 16 (12.4%) | 0 (0%) | 0.254 |
| Italy | 32 (24.8%) | 4 (80.0%) |  |
| Norway | 12 (9.3%) | 0 (0%) |  |
| Spain | 10 (7.8%) | 0 (0%) |  |
| The Netherlands | 45 (34.9%) | 1 (20.0%) |  |
| United Kingdom | 4 (3.1%) | 0 (0%) |  |
| Belgium | 10 (7.8%) | 0 (0%) |  |
| Gender |  |  |  |
| Female | 46 (35.7%) | 2 (40.0%) | 1 |
| Male | 83 (64.3%) | 3 (60.0%) |  |
| Age(years) |  |  |  |
| Mean (SD) | 7.17 (5.14) | 20.7 (1.12) | *P*<0.001 |
| Median [Min, Max] | 5.60 [0.300, 17.8] | 20.8 [19.3, 21.8] |  |
| Site of primary tumor |  |  |  |
| Extremities | 12 (9.3%) | 0 (0%) | 0.102 |
| GUBP | 23 (17.8%) | 1 (20.0%) |  |
| GUnoBP | 2 (1.6%) | 1 (20.0%) |  |
| HNnoPM | 17 (13.2%) | 0 (0%) |  |
| HNPM | 44 (34.1%) | 3 (60.0%) |  |
| Orbit | 17 (13.2%) | 0 (0%) |  |
| Other site | 14 (10.9%) | 0 (0%) |  |
| Site of primary tumor |  |  |  |
| Favourable | 37 (28.7%) | 1 (20.0%) | 1 |
| Unfavourable | 92 (71.3%) | 4 (80.0%) |  |
| Histology |  |  |  |
| Alveolar | 26 (20.2%) | 3 (60.0%) | 0.1 |
| Embryonal | 94 (72.9%) | 2 (40.0%) |  |
| Other | 9 (7.0%) | 0 (0%) |  |
| Fusion status |  |  |  |
| Missing | 32 (24.8%) | 3 (60.0%) | 0.161 |
| Negative | 77 (59.7%) | 1 (20.0%) |  |
| Positive | 20 (15.5%) | 1 (20.0%) |  |
| Tumor size |  |  |  |
| <=5 cm | 67 (51.9%) | 3 (60.0%) | 1 |
| >5 cm | 62 (48.1%) | 2 (40.0%) |  |
| T |  |  |  |
| T0 | 1 (0.8%) | 0 (0%) | 0.466 |
| T1 | 61 (47.3%) | 1 (20.0%) |  |
| T2 | 67 (51.9%) | 4 (80.0%) |  |
| N |  |  |  |
| N0 | 91 (70.5%) | 3 (60.0%) | 0.994 |
| N1 | 38 (29.5%) | 2 (40.0%) |  |
| M |  |  |  |
| M0 | 104 (80.6%) | 3 (60.0%) | 0.576 |
| M1 | 25 (19.4%) | 2 (40.0%) |  |
| Risk group |  |  |  |
| Standard | 46 (35.7%) | 1 (20.0%) | 0.636 |
| High | 49 (38.0%) | 2 (40.0%) |  |
| Very high - localized | 9 (7.0%) | 0 (0%) |  |
| Very high - metastatic | 25 (19.4%) | 2 (40.0%) |  |
| Event |  |  |  |
| No | 85 (65.9%) | 2 (40.0%) | 0.476 |
| Yes | 44 (34.1%) | 3 (60.0%) |  |
| Status at last follow-up |  |  |  |
| Alive off therapy (1st CR) | 82 (63.6%) | 2 (40.0%) | 0.543 |
| Alive off therapy (2nd CR) | 10 (7.8%) | 0 (0%) |  |
| Alive with disease | 2 (1.6%) | 0 (0%) |  |
| Dead | 33 (25.6%) | 3 (60.0%) |  |
| Lost to follow-up in CR | 2 (1.6%) | 0 (0%) |  |

| Suppl. Material 5. Baseline characteristics of the pediatric/adolescent versus young adult cohort in the excluded and included cohort | | | | |
| --- | --- | --- | --- | --- |
|  | Excluded | | Included | |
|  | Pediatric/Adolescent (*n*=48) | Young adult (*n*=4) | Pediatric/Adolescent (*n*=81) | Young adult (*n*=1) |
| Country |  |  |  |  |
| France | 3 (6.3%) | 0 (0%) | 13 (16.0%) | 0 (0%) |
| Italy | 11 (22.9%) | 3 (75.0%) | 21 (25.9%) | 1 (100%) |
| Norway | 6 (12.5%) | 0 (0%) | 6 (7.4%) | 0 (0%) |
| Spain | 8 (16.7%) | 0 (0%) | 2 (2.5%) | 0 (0%) |
| The Netherlands | 17 (35.4%) | 1 (25.0%) | 28 (34.6%) | 0 (0%) |
| United Kingdom | 1 (2.1%) | 0 (0%) | 3 (3.7%) | 0 (0%) |
| Belgium | 2 (4.2%) | 0 (0%) | 8 (9.9%) | 0 (0%) |
| Gender |  |  |  |  |
| Female | 24 (50.0%) | 2 (50.0%) | 22 (27.2%) | 0 (0%) |
| Male | 24 (50.0%) | 2 (50.0%) | 59 (72.8%) | 1 (100%) |
| Age(years) |  |  |  |  |
| Mean (SD) | 7.01 (4.89) | 20.4 (1.07) | 7.26 (5.32) | 21.8 (NA) |
| Median [Min, Max] | 5.05 [0.600, 16.9] | 20.3 [19.3, 21.7] | 5.80 [0.300, 17.8] | 21.8 [21.8, 21.8] |
| Site of primary tumor |  |  |  |  |
| Extremities | 6 (12.5%) | 0 (0%) | 6 (7.4%) | 0 (0%) |
| GUBP | 6 (12.5%) | 0 (0%) | 17 (21.0%) | 1 (100%) |
| HNnoPM | 7 (14.6%) | 0 (0%) | 10 (12.3%) | 0 (0%) |
| HNPM | 15 (31.3%) | 3 (75.0%) | 29 (35.8%) | 0 (0%) |
| Orbit | 8 (16.7%) | 0 (0%) | 9 (11.1%) | 0 (0%) |
| Other site | 6 (12.5%) | 0 (0%) | 8 (9.9%) | 0 (0%) |
| GUnoBP | 0 (0%) | 1 (25.0%) | 2 (2.5%) | 0 (0%) |
| Site of primary tumor |  |  |  |  |
| Favourable | 15 (31.3%) | 1 (25.0%) | 22 (27.2%) | 0 (0%) |
| Unfavourable | 33 (68.8%) | 3 (75.0%) | 59 (72.8%) | 1 (100%) |
| Histology |  |  |  |  |
| Alveolar | 12 (25.0%) | 3 (75.0%) | 14 (17.3%) | 0 (0%) |
| Embryonal | 31 (64.6%) | 1 (25.0%) | 63 (77.8%) | 1 (100%) |
| Other | 5 (10.4%) | 0 (0%) | 4 (4.9%) | 0 (0%) |
| Fusion status |  |  |  |  |
| Missing | 13 (27.1%) | 2 (50.0%) | 19 (23.5%) | 1 (100%) |
| Negative | 26 (54.2%) | 1 (25.0%) | 51 (63.0%) | 0 (0%) |
| Positive | 9 (18.8%) | 1 (25.0%) | 11 (13.6%) | 0 (0%) |
| Tumor size |  |  |  |  |
| <=5 cm | 29 (60.4%) | 2 (50.0%) | 38 (46.9%) | 1 (100%) |
| >5 cm | 19 (39.6%) | 2 (50.0%) | 43 (53.1%) | 0 (0%) |
| T |  |  |  |  |
| T0 | 1 (2.1%) | 0 (0%) | 0 (0%) | 0 (0%) |
| T1 | 30 (62.5%) | 1 (25.0%) | 31 (38.3%) | 0 (0%) |
| T2 | 17 (35.4%) | 3 (75.0%) | 50 (61.7%) | 1 (100%) |
| N |  |  |  |  |
| N0 | 34 (70.8%) | 2 (50.0%) | 57 (70.4%) | 1 (100%) |
| N1 | 14 (29.2%) | 2 (50.0%) | 24 (29.6%) | 0 (0%) |
| M |  |  |  |  |
| M0 | 38 (79.2%) | 2 (50.0%) | 66 (81.5%) | 1 (100%) |
| M1 | 10 (20.8%) | 2 (50.0%) | 15 (18.5%) | 0 (0%) |
| Risk group |  |  |  |  |
| Standard | 19 (39.6%) | 1 (25.0%) | 27 (33.3%) | 0 (0%) |
| High | 17 (35.4%) | 1 (25.0%) | 32 (39.5%) | 1 (100%) |
| Very high - localized | 2 (4.2%) | 0 (0%) | 7 (8.6%) | 0 (0%) |
| Very high - metastatic | 10 (20.8%) | 2 (50.0%) | 15 (18.5%) | 0 (0%) |
| Event |  |  |  |  |
| No | 34 (70.8%) | 2 (50.0%) | 51 (63.0%) | 0 (0%) |
| Yes | 14 (29.2%) | 2 (50.0%) | 30 (37.0%) | 1 (100%) |
| Status at last follow-up |  |  |  |  |
| Alive off therapy (1st CR) | 32 (66.7%) | 2 (50.0%) | 50 (61.7%) | 0 (0%) |
| Alive off therapy (2nd CR) | 5 (10.4%) | 0 (0%) | 5 (6.2%) | 0 (0%) |
| Alive with disease | 1 (2.1%) | 0 (0%) | 1 (1.2%) | 0 (0%) |
| Dead | 9 (18.8%) | 2 (50.0%) | 24 (29.6%) | 1 (100%) |
| Lost to follow-up in CR | 1 (2.1%) | 0 (0%) | 1 (1.2%) | 0 (0%) |

| Suppl. Material 6. Comparison for pediatric/adolescent versus included cohort | | | |  |
| --- | --- | --- | --- | --- |
| Variable | Pediatric/adolescent (*n*=81) | Included cohort (*n*=82) | *P*-value | |
| Mean ADC diagnosis | 1.123 | 1.129 | 0.90 | |
| Mean ADC response | 1.556 | 1.553 | 0.96 | |
| Mean ADC absolute change | 0.433 | 0.424 | 0.89 | |
| Mean ADC percental change | 44.98 % | 44.23 % | 0.91 | |
| ADC: Apparent Diffusion Coefficient | | | |  |

| Suppl. Material 7. Univariable Cox proportional hazard analysis for event-free survival for patients with similar scanner properties at diagnosis and response (*n*=38) | | | |
| --- | --- | --- | --- |
| Variable |  | Hazard ratio | (95% CI ) |
| ADC 5^th^ percentile | absolute change | 0.83 | (0.24 – 2.9) |
| Mean ADC | absolute change | 0.79 | (0.19 – 3.2) |
| ADC 5^th^ percentile | diagnosis | 0.4 | (0.05 – 3.0) |
| Mean ADC | diagnosis | 0.43 | (0.05 – 3.9) |
| ADC 5^th^ percentile | response | 0.62 | (0.19 – 2.0) |
| Mean ADC | response | 0.5 | (0.1 – 2.4) |
| ADC: Apparent Diffusion Coefficient; CI: Confidence Interval. | | | |
|  |  |  |  |

| Suppl. Material 8. Events in all patients and patients included for ADC analyses | | |
| --- | --- | --- |
|  | All patients  (*n*=134) | Included patients for ADC analyses  (*n=82)* |
| Event |  |  |
| Local relapse | 18 (13.4%) | 13 (15.9%) |
| Progressive disease | 11 (8.2%) | 5 (6.1%) |
| Metastatic relapse | 16 (11.9%) | 11 (13.4%) |
| Local and metastatic relapse | 2 (1.5%) | 2 (2.4%) |
| Toxic death | 0 (0%) | 0 (0%) |
| No event | 87 (64.9%) | 51 (62.2%) |
|  | | |
